# Supplementary material for: Deterministic and highly indistinguishable single photons in the telecom C-band
Source: Nat Commun. 2026 Jan 14;17:537. doi: 10.1038/s41467-026-68336-0 (PMC12804856; doi:10.1038/s41467-026-68336-0)
Supplement: Supplementary file 1 — Supplementary Information [file 41467_2026_68336_MOESM1_ESM.pdf]

Supplementary Information
Deterministic and highly indistinguishable single photons in the telecom C-band

Nico Hauser[1,*], Matthias Bayerbach[1], Jochen Kaupp[2], Yorick Reum[2], Giora Peniakov[2], Johannes Michl[2], Martin Kamp[2], Tobias Huber-Loyola[2], Andreas T. Pfenning[2], Sven Höfling[2], and Stefanie Barz[1]
[1]*Institute for Functional Matter and Quantum Technologies and Center for Integrated Quantum Science and Technology (IQST), University of Stuttgart, 70569 Stuttgart, Germany*
[2]*Julius-Maximilians-Universität Würzburg, Physikalisches Institut, Lehrstuhl für Technische Physik, 97074 Würzburg, Germany*
[*]*nico.hauser@fmq.uni-stuttgart.de*

A) Pump wavelength sweep

By sweeping the wavelength of the pump laser and simultaneously measuring the QD emission, we can identify resonances to optically excite the QD. The results of the wavelength sweep are presented in the main manuscript. Two strong resonances have been identified at $\lambda_{\text{pump}} = 1404.2\,\text{nm}$ (resonance #1) and $\lambda_{\text{pump}} = 1498.2\,\text{nm}$ (resonance #2).

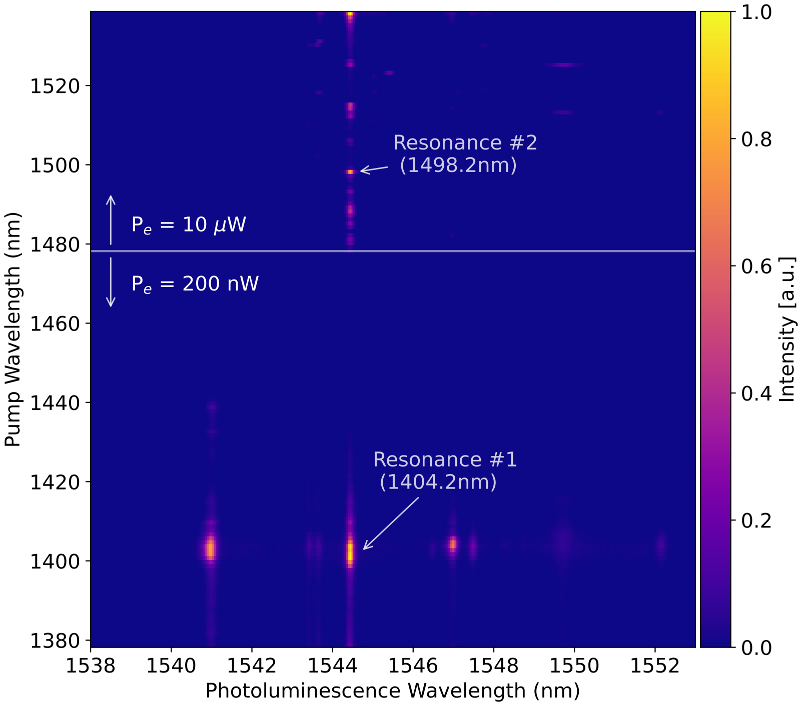

Supplementary Figure 1: **Photoluminescence spectrum of the InAs/InAlGaAs quantum dot during a wavelength sweep of the excitation laser.** The wavelength of the pump laser was tuned between $\lambda_{\text{pump}} = 1378.0\,\text{nm}$ and $\lambda_{\text{pump}} = 1538.0\,\text{nm}$ in steps of 1.0 nm. For $\lambda_{\text{pump}} < 1478.0\,\text{nm}$ a pump power of $P_{\text{exc}} = 200\,\text{nW}$ was used to avoid saturation of the emission in this regime. The pump power was increased to $P_{\text{exc}} = 10\,\mu\text{W}$ for $\lambda_{\text{pump}} \geq 1478.0\,\text{nm}$ to account for the lower excitation efficiency of the observed emission. Note that for the measurement in this regime an additional 12 nm FWHM bandpass filter was used to avoid pump light saturating the spectrometer, hence removing the emission line at 1541 nm. Bright resonances were identified at $\lambda_{\text{pump}} = 1404.2\,\text{nm}$ (resonance #1) and $\lambda_{\text{pump}} = 1498.2\,\text{nm}$ (resonance #2).

B) Blinking

By repeating the measurement of the second-order correlation function and integrating over longer time periods, blinking can be observed as depicted in Supplementary Figure 2. The blinking behaviour can be modeled as [1, 2]

$$A_{\mathrm{n}}(t) = A_0 \left(1 + A \exp - \frac{|t|}{\tau_{\mathrm{B}}}\right), \tag{1}$$

where A_n corresponds to the area underneath the nth coincidence peak. Here, τ_{B} is the blinking time, A the blinking strength and A_0 the coincidence peak area for $t >> \tau$. By fitting $A_n(t)$ in Eqn. (1) to the data presented in Supplementary Figure 2, we obtain a blinking strength of $A = 2.71 \pm 0.01$ and a blinking time of $\tau_{\mathrm{B}} = (294 \pm 2)$ns.

From the blinking behaviour, we can estimate a blinking-related efficiency η_{Blink} as [3]

$$\eta_{\mathrm{Blink}} = \frac{1}{1 + A}, \tag{2}$$

which for the measured QD corresponds to $\eta_{\mathrm{Blink}} = (26.9 \pm 0.1)\%$.

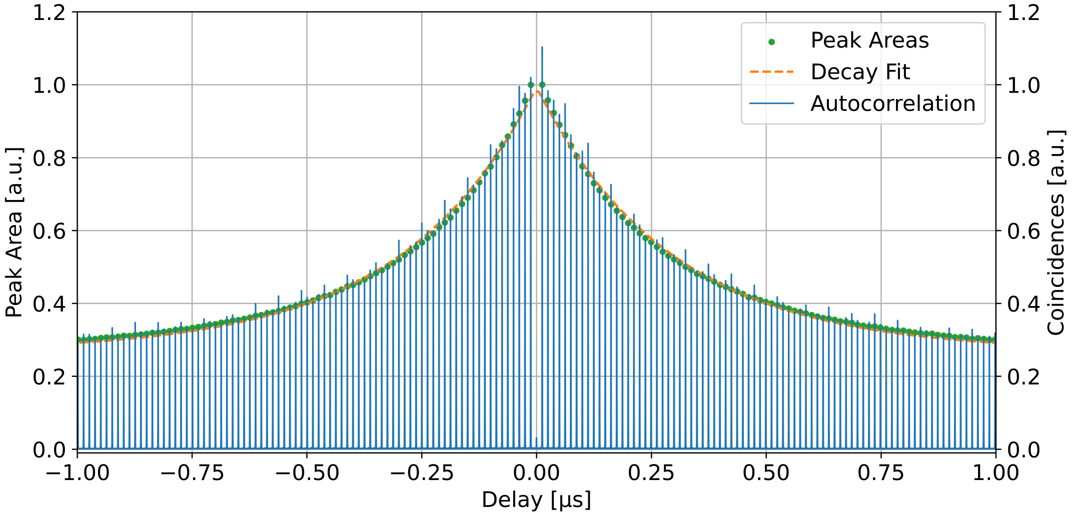

Supplementary Figure 2: **Emission characteristics of the investigated QD on μs timescales.** Autocorrelation of the QD emission measured using an HBT-setup. Significant blinking with a strength of $A = 2.79 \pm 0.02$ at a timescale of $\tau = (291 \pm 3)$ ns can be observed when using LA-phonon-assisted excitation.

C) Source Efficiency Estimation

An important metric of single-photon sources is the brightness [4, 5]. When exciting the QD with a repetition rate of 80 MHz, we measure a rate of 400k photons per second on the SNSPDs for LA-phonon-assisted excitation when the emission is saturated at $P_{\mathrm{Laser}} = 1.5\,\mu$W. This corresponds to an overall efficiency of 0.5%.
By characterizing the losses of elements in the setup, we can infer the corrected setup efficiency. The known losses are listed in Supplementary Table I and add up to a total loss of 76% caused by lossy optical elements and non-unity detector efficiency. Correcting for these losses, we estimate a corrected overall efficiency of 2.1%. The overall efficiency is currently limited by factors such as residual blinking, suboptimal coupling of QD photons into the single-mode fiber, and non-ideal photon extraction from the CBG. Addressing these aspects offers clear pathways for further improvement in efficiency.

Element	Efficiency
90:10 BS	0.88
Cryostat Window	0.98
12 nm Bandpass	0.97
Variable Bandpass (0.1 nm)	0.36
Beam sampler for camera	0.93
Silver mirrors (4x)	0.93
Fibers & Connectors	0.96
Detector	0.94
Total efficiency	0.24

Supplementary Table I: **Losses in the QD setup.** Losses were measured at 1550 nm or given by the supplier of the elements.

D) Lifetime Measurements

To assess the temporal behavior of the QD emission under the various excitation schemes, we perform time-resolved single-photon measurements. In order to gain the temporal information, we measure the photons' arrival time at the SNSPDs with respect to the clock signal of the pump laser. Additionally, we estimate the instrument response function of our detection system by performing the same measurement with reference laser pulses ($\Delta\tau = 2$ ps). We clearly observe the fastest dynamics for LA-phonon-assisted excitation, which is in good agreement with the high two-photon indistinguishability measured for this excitation scheme. We assume that the slower dynamics observed for above-band-gap excitation, resonance #1 and resonance #2 originate from slower relaxation channels into the excited state.

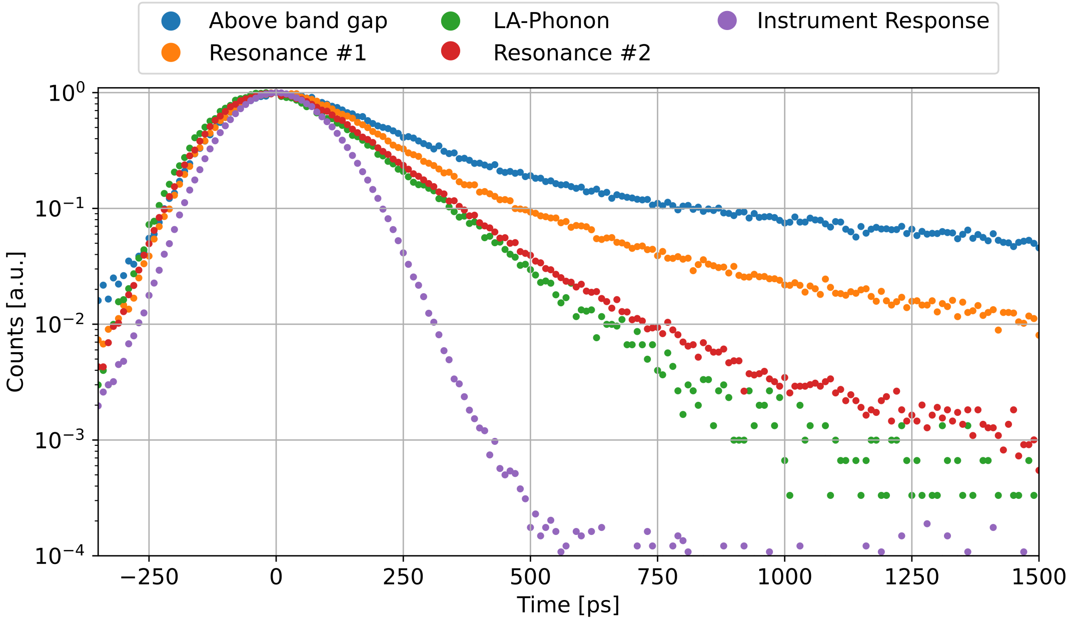

Supplementary Figure 3: **Time-resolved single-photon measurements for the different QD excitation schemes.** The temporal behaviour of the QD emission is investigated through the exponential decay in photon counts. Here, we clearly observe different timescales with above-band-gap excitation inhibiting the slowest, and LA-phonon-assisted excitation the fastest dynamics. Additionally, the instrument response is measured using pump laser pulses as reference.

E) Detailed Experimental Setup

A detailed sketch of the experimental setup is shown in Supplementary Figure 4. The figure is divided into two parts: Photon generation (highlighted in blue) and Measurement apparatus (highlighted in green). The photon generation consists of optical elements required for pump-pulse preparation, QD-sample imaging, single photon extraction and spectral filtering. The measurement apparatus depicts the active demultiplexing, delay stage, 50:50 BS and SNSPDs with coincidence logic. This sketch serves as an extension of the simplified experimental setup presented in Fig. 1 of the main text.

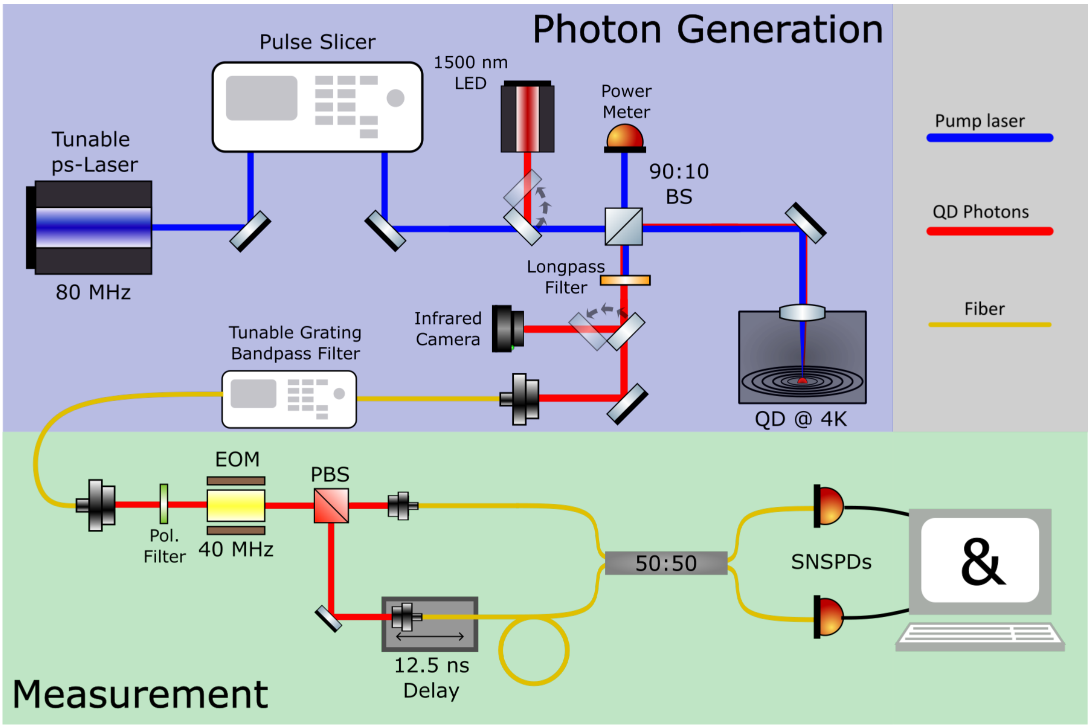

Supplementary Figure 4: **Detailed sketch of the experimental setup.** Here, pulse slicing, sample illumination and imaging are included in the sketch to depict the full scope of the experimental apparatus.

F) Logarithmic Correlation Plots

In order to more intuitively assess certain features of the measured correlations, we additionally depict the correlation data from the main manuscript with logarithmic y-axis scaling in Supplementary Figure 5 and 6. Here, the different timescales of the excitation dynamics can clearly be identified with above band gap excitation showing the slowest and LA-phonon assisted excitation the fastest dynamics. Furthermore, the additional side peaks at a delay of ±12.5 ns and ±37.5 ns can clearly be resolved. These originate from the imperfect switching extinction of the employed EOMs depicted in Supplementary Figure 4 b and c.

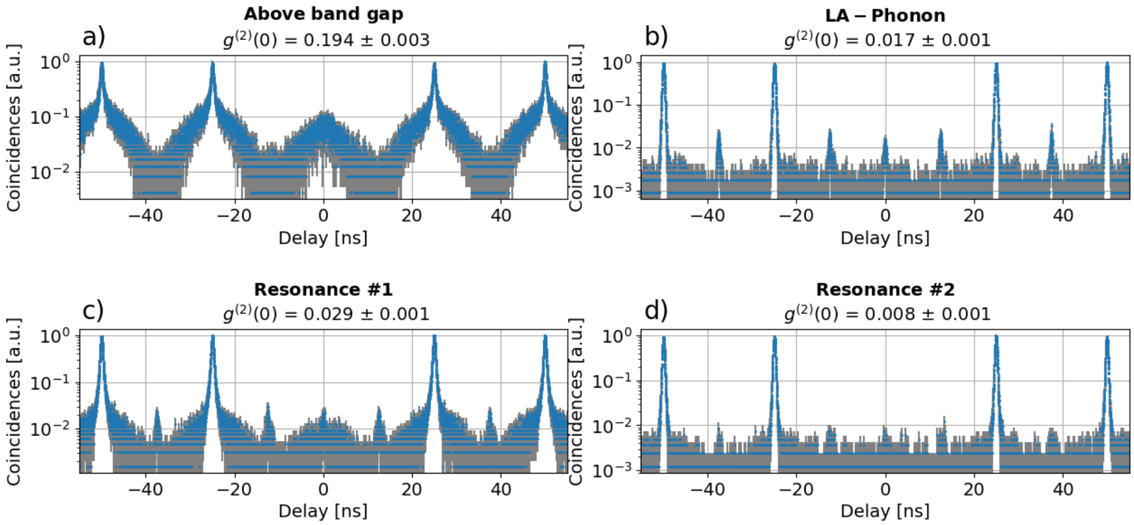

Supplementary Figure 5: **Measurement of photon statistics for different excitation schemes with logarithmic y-axis scaling.**

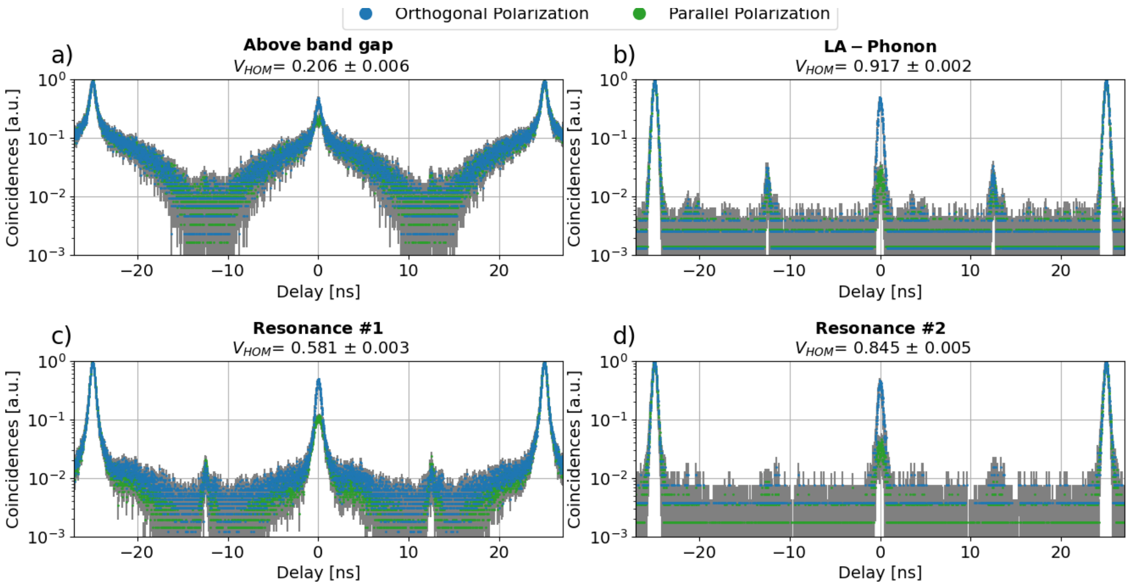

Supplementary Figure 6: **Two-photon interference measurements for different excitation schemes with logarithmic y-axis scaling.**

REFERENCES

[1] Santori, C., Pelton, M., Solomon, G., Dale, Y. & Yamamoto, Y. Triggered single photons from a quantum dot. *Phys. Rev. Lett.* **86**, 1502–1505 (2001).

[2] Kim, J. *et al.* Two-photon interference from an inas quantum dot emitting in the telecom c-band (2025).

[3] Vajner, D. A. *et al.* On-demand generation of indistinguishable photons in the telecom c-band using quantum dot devices. *ACS Photonics* **11**, 339–347 (2024).

[4] Liu, J. *et al.* A solid-state source of strongly entangled photon pairs with high brightness and indistinguishability. *Nature nanotechnology* **14**, 586–593 (2019).

[5] Wang, H. *et al.* On-demand semiconductor source of entangled photons which simultaneously has high fidelity, efficiency, and indistinguishability. *Physical review letters* **122**, 113602 (2019).
